# Effectiveness of physiotherapy interventions for back care and the prevention of non-specific low back pain in children and adolescents: a systematic review and meta-analysis

**DOI:** 10.1186/s12891-022-05270-4

**Published:** 2022-04-02

**Authors:** José Manuel García-Moreno, Inmaculada Calvo-Muñoz, Antonia Gómez-Conesa, José Antonio López-López

**Affiliations:** 1 International School of Doctoral Studies, University of Murcia, Murcia, Spain; 2 Department of Physiotherapy, UCAM Catholic University of Murcia, Murcia, Spain; 3 Research Group Research Methods and Evaluation in Social Sciences. Mare Nostrum Campus of International Excellence, University of Murcia, Murcia, Spain; 4 Department of Basic Psychology and Methodology, University of Murcia, Murcia, Spain

**Keywords:** Physical therapy modalities, Primary prevention, Child, Adolescent, Meta-analysis, Low back pain, Exercise

## Abstract

**Background:**

Non-specific low back pain in children and adolescents has increased in recent years. The purpose of this study was to upgrade the evidence of the most effective preventive physiotherapy interventions to improve back care in children and adolescents.

**Methods:**

The study settings were children or adolescents aged 18 years or younger. Data were obtained from the Cochrane Library, MEDLINE, PEDro, Web of Science, LILACS, IBECS, and PsycINFO databases and the specialized journals BMJ and Spine. The included studies were published between May 2012 and May 2020. Controlled trials on children and adolescents who received preventive physiotherapy for back care were considered. Data on all the variables gathered in each individual study were extracted by two authors separately. Two authors assessed risk of bias of included studies using the RoB2 and quality of the body of evidence using the GRADE methodology. Data were described according to PRISMA guidelines. To calculate the effect size, a standardized mean difference “d” was used and a random-effects model was applied for the following outcome variables: behaviour, knowledge, trunk flexion muscle endurance, trunk extension muscle endurance, hamstring flexibility and posture.

**Results:**

Twenty studies were finally included. The most common physiotherapy interventions were exercise, postural hygiene and physical activity. The mean age of the total sample was 11.79 years. When comparing the change from baseline to end of intervention in treatment and control groups, the following overall effect estimates were obtained: behaviour d_+_ = 1.19 (95% CI: 0.62 and 1.76), knowledge d_+_ = 1.84 (0.58 and 3.09), trunk flexion endurance d_+_ = 0.65 (-0.02 and 1.33), trunk extension endurance d_+_ = 0.71 (0.38 and 1.03), posture d_+_ = 0.65 (0.24 and 1.07) and hamstrings flexibility d_+_ = 0.46 (0.36 and 0.56). At follow-up, the measurement of the behaviour variable was between 1 and 12 months, with an effect size of d_+_ = 1.00 (0.37 and 1.63), whereas the knowledge variable obtained an effect size of d_+_ = 2.08 (-0.85 and 5.02) at 3 months of follow-up.

**Conclusions:**

Recent studies provide strong support for the use of physiotherapy in the improvement of back care and prevention of non-specific low back pain in children and adolescents. Based on GRADE methodology, we found that the evidence was from very low to moderate quality and interventions involving physical exercise, postural hygiene and physical activity should be preferred.

**Supplementary information:**

The online version contains supplementary material available at 10.1186/s12891-022-05270-4.

## Background

Low back pain (LBP) is a public health problem and the prevalence of LBP in children and adolescents has increased in recent years [1, 2], reaching around 39% of LBP lifetime prevalence in 9 to 16 years of age with a similar prevalence to adults at 15 years of age [3]. The presence of LBP in childhood and adolescence increases the risk of suffering it in adulthood [2, 4]. Non-specific low back pain (NSLBP) is the most common type of LBP [1], therefore it is important to rule out the presence of spondylolysis and spondylolisthesis which may cause LBP [2].

Although strategies for back care and NSLBP prevention have been examined in less detail in children and adolescents than in adult populations [5], different approaches from physiotherapy can be found in the literature [6]. One of them arises from the fact that adolescents [7–9] and parents [10] generally do not have sufficient knowledge about back care and hence different methods have been developed to fill this gap [6]. This knowledge can be taught through the application of postural hygiene (theory or practice) and through physical exercise aimed at establishing knowledge of back care. Having adequate knowledge about back care can give children and adolescents the ability to change their lifestyles on their own [8].

Another way to promote back care is through behaviour change in daily activities in which the back may be affected [11–13], including the correct use of the schoolbags and limiting their weight to 10-15% of the child’s weight [10], changing posture evenly [11], lifting weights off the floor appropriately [12], and improving sitting and standing postures for prolonged periods [11, 12]. As well as knowledge, behaviour can be taught through postural hygiene and physical exercise associated with back care in order to establish the concepts learned [11–13].

Besides, other methods used in physiotherapy to improve back care and prevent NSLBP in children and adolescents include improving the strength of the trunk muscles through specific exercises [14], for this, the exercises must be ordered and supervised by a professional, should be done progressively, and can be practiced by both children and adolescents [15]. Increasing hamstring flexibility is also a good way to improve back care [16].

In recent years, some systematic reviews have been published on this topic, some have focused on a single variable such as posture [17, 18], some included articles only in the physical education field [19] or some included several variables such as knowledge and behaviour without quantitative analysis [12, 20]. In a previous meta-analysis in 2012 we analyzed the effects of preventive physiotherapy treatments on knowledge and behaviour [21] and since then no meta-analysis has been carried out that quantified the effects of physiotherapy treatments on back care in children and adolescents.

Considering the heterogeneity of the procedures to improve back care in children and adolescents, the numerous clinical trials that have been published on this topic in recent years, and the fact that no recent meta-analyses have been carried out that encompass them, an objective analysis of the effects of these preventive procedures is necessary.

Therefore, this systematic review aimed to find out which preventive physiotherapy interventions are most effective to improve back care and to prevent non-specific low back pain in children and adolescents.

## Methods

### Study design

This meta-analysis was carried out following the Preferred Reporting Items for Systematic Reviews and Meta-analyses (PRISMA) statement guidelines [22] and registered with PROSPERO (CRD42021236645).

### Eligibility criteria

We included randomized controlled trials (RCTs), cluster RCTs, and quantitative controlled quasi-experimental studies if they reported a pre-test and post-test evaluation. Studies had to be published or finished between May 2012 (the end date of the search period of the latest meta-analysis on this topic) and May 2020. Case, cohort, and uncontrolled studies were excluded. No language restrictions were applied. The participants had to be children or adolescents aged 18 years or younger. Studies in which all subject in the sample presented LBP, spinal diseases, surgical vertebral treatment or other pathologies that cause LBP were excluded. The studies had to apply a back care physical therapy or preventive treatment for NSLBP without pharmacological treatment (postural hygiene, exercise, physical activity or others). The control groups could be active or non-active. The results had to be collected with the same tool with which the initial evaluations were collected and the studies had to report enough statistical data to calculate the effect sizes.

### Data sources

Several methods were used to search for studies: different specialized databases, specialized journals on the subject, and the bibliography of expert authors on the subject were searched. Published and unpublished articles were searched.

The different specialized databases were Cochrane Library, MEDLINE, PEDro, Web of Science (WoS), LILACS, IBECS, and PsycINFO and the specialized journals were BMJ and Spine. In the search for unpublished articles, articles by relevant authors, conference acts, and doctoral theses were examined. Besides, the bibliography of articles already collected and relevant articles were searched.

### Search strategy

The searches were carried out from February to May 2020, with a combination of the following keywords: adolescent, child, young, school, “back pain”, “low back pain”, “back complaint”, “back care”, prevention, education, “postural hygiene”, “physical education”, “back education”, “posture education”, “back function”, physiotherapy, backpack, ergonomics, “physical therapy”, “exercise therapy”, promotion, knowledge, behaviour and “cognitive behavioural therapy”.

For more details about the search terms and combinations, see Additional file 1.

The search was conducted by one author (JGM) and all researchers jointly decided which studies should be included.

### Data extraction

The data extraction of the articles was carried out based on a previously exposed coding manual. In this manual, according to Lipsey’s recommendations [23] the variables have been grouped into three different categories: substantive (treatment, context, and participant), methodological, and extrinsic variables. For more information about the coded variables, see Additional file 2.

Data on all the variables gathered in each individual study were extracted by two authors separately (JGM, ICM). To resolve discrepancies between the two authors, a third author (AGC) intervened to decide the extracted data. When required, additional data were requested directly from the corresponding authors.

In order to assess the reliability of the coding process, Cohen’s Kappa was calculated for qualitative variables and the intraclass correlation coefficient (ICC) for quantitative variables [24]. Kappa values ranged between 0.827 and 1 and the ICC ranged from 0.993 to 1.

### Risk of bias assessment

The Cochrane Risk of Bias 2 tool, which is designed for clinical trials, was used to calculate the risk of bias (RoB). The RoB was independently examined in the same way as data extraction. This tool allows a clinical trial to be assessed as “low RoB”, “some concerns” or “high RoB”.

To determine the RoB of an article, the worst judgment from all domains was chosen. In case that the evaluation had “some concerns” in several domains, it was rated at “high RoB” as recommended by the authors [25].

The RoB was also assessed by the same researchers who coded the variables (JGM and ICM). Cohen’s Kappa was used to assess inter-rater agreement, with a result of 1.

### Type of outcome measures

The studies had to have at least one outcome related to back care. Outcomes included were back care behaviour, back care knowledge, trunk flexion endurance, trunk extension endurance, hamstrings flexibility, posture, lower limb power, awareness, sitting time, standing time, stepping, step counts, sit-to-stand counts, upper limbs muscular endurance, cardiovascular endurance, lumbar motor control, skills, self-efficacy, and beliefs.

### Effect size index

To calculate the effect size, a standardized mean difference *“d”* was used [26] for quantitative variables [27]. To calculate the effect size concerning follow-up, the same procedure was followed, replacing the post-test data with the follow-up data [28]. The magnitude of d indices is sometimes interpreted following Cohen’s tentative benchmarks: 0 null, ± 0.20 low, ± 0.50 medium, ± 0.80 high [29]. The effect size was calculated by the first author (JGM) with the supervision of another researcher (JLL).

### Data analysis

A random-effects model was applied for each outcome variable reported in at least two studies [26] using the correction proposed by Hartung [30]. A forest plot with 95% confidence intervals was created to represent numerically and graphically the individual effects of each study, in addition to representing the average effect size. Forest plots were created for all meta-analyzed variables, namely: behaviour, knowledge, posture, hamstring flexibility, trunk flexion endurance and trunk extension endurance. To assess heterogeneity, the I^2^ index was used. To help with interpretation of the I^2^ values, the tentative benchmarks of 25% low, 50% medium, and 75% high may be followed.

To analyze moderator variables, weighted ANOVA was used for the qualitative moderator variables and meta-regression for the continuous moderator variables. Both analyses were corrected as proposed by Knapp and Hartung [31].

All statistical analyses were performed using R [32] in conjunction with the metafor package [33]. The PRISMA checklist was used to check the reporting quality of the meta-analysis [22] (Additional file 3).

## Results

**Fig. 1:**
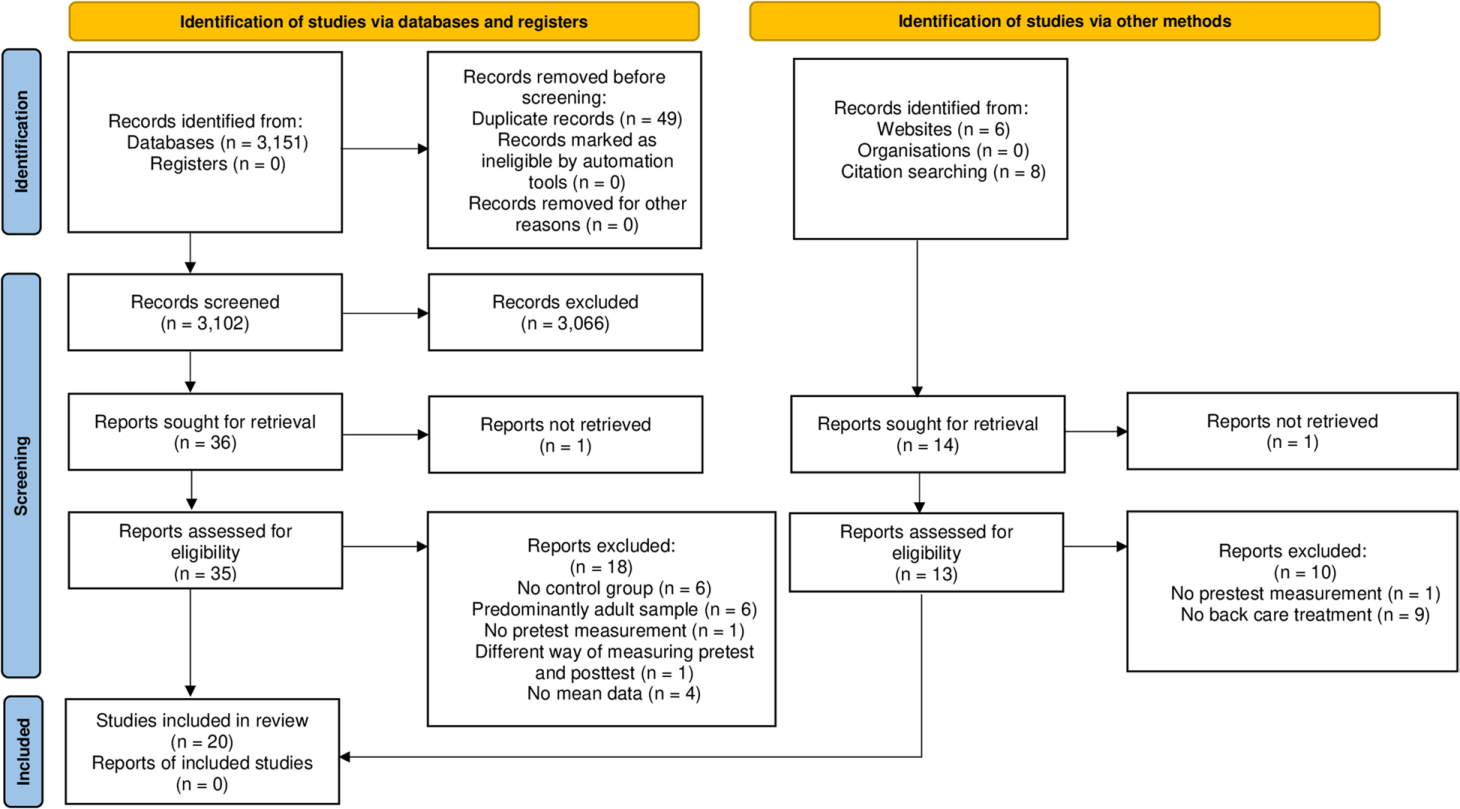
PRISMA flow diagram. Process of identification and selection of studies

A total of 3166 references were located, of which 3151 were found in the databases and 14 in the search in other sources. After removing duplicates and conducting a first analysis of the studies, we were left with 50. The main reasons we eliminated those studies were because the participants were adults, the participants had pathologies that caused LBP, and that the treatment included pharmacotherapy. Finally, the rest of the papers were screened at full text, and 20 papers met the inclusion criteria. All papers compared an experimental group and a control group. After the identification process, twenty articles were selected [34–53]. Figure 1 shows the process of identification and selection of the studies.

Other studies that appear to meet the inclusion criteria were excluded because there was no control group [14], no pretest measurement [54], no mean data [55], and no variables related to back care [56].

### Study characteristics

The included studies were published between 2012 and 2020, all published in journals except for one conference presentation [38]. Eleven articles were RCTs [36, 37, 39, 41, 43, 46–50, 53] and nine were non-randomized controlled trials [34, 35, 38, 40, 42, 44, 45, 51, 52]. A cluster randomization were applied for all the RCT except one [50]. All the studies were carried out in the school except one that was carried out in a sport center [50] and one is not specified [38]. Six studies were carried out in Spain [39, 41, 42, 48, 49, 51], four in Brazil [35, 37, 44, 52], two in South Africa [43, 47], two in Iran [36, 53], two in Germany [38, 46], and one in Turkey [34], New Zealand [40], Poland [45] and Hungary [50]. The first author of ten studies was a physical education teacher [34, 35, 38, 40–42, 44, 48, 49, 51] and in seven was a physiotherapist [37, 39, 43, 45, 47, 50, 53].

The sample size in the pretest of all the participants of the experimental groups was 1,546 and the sample size of the control groups was 1,315. In the posttest, the sample size of the experimental groups was 1,374 and the sample size of the control groups was 1,263. In the follow-up, the sample size of the experimental groups was 675 and the sample size of the control groups was 493, this is due to only seven studies report follow-up data [36, 39, 43, 44, 47, 51, 53].

Regarding the type of intervention of the experimental groups, the most common was exercise, carried out in fifteen studies [34, 35, 37, 38, 41, 42, 44, 46–53], followed by postural hygiene, carried out in nine studies [36, 39, 43–47, 51, 53], physical activity, carried out in six studies [34, 41, 44, 50–52] and standing workstations in classroom, carried out in one study [40]. Exercise, postural hygiene and physical activity were combined in some studies. Regarding the weeks of the intervention were from 1 to 24, the intensity was from 0.31 to 6.41 h and the magnitude was from 0.75 to 77 h. The number of sessions of the experimental group were established before the start of the study in all of the studies except two [38, 40] and the treatment of all experimental groups were homogeneous except in one study [38]. Thirteen studies were conducted with children [34–37, 39–41, 43, 45, 46, 51–53] and seven with adolescents [38, 42, 44, 47–50]. The mean age of the participants in the experimental group was from 7.6 to 15.22 years with a mean of 11.73 years and in the control group was from 7.72 to 15.55 years with a mean of 11.85 years, the mean age of all participants was 11.79 years. The percentage of males in the experimental group was from 31.17 to 74% and in the control group was from 34.08 to 73.58%, all the studies include boys and girls in their samples except one that only participated girls [53]. Concerning the control groups, in seven studies the control group was active [35, 38, 41, 42, 48, 49, 51] and the rest was inactive. For more information, see Additional file 4.

### Risk of bias

Nine studies were considered to report results with high RoB [34, 35, 38, 40, 42, 44, 45, 51, 52] whereas some concerns were found for eleven studies [36, 37, 39, 41, 43, 46–50, 53]. Concerns regarding randomization led to high RoB assessments in eight studies. In the second domain, all the studies had some concerns, since the therapists knew the group they were treating and blinding of participants was not possible due to the nature of the treatment. All except two studies [35, 52] were assessed at RoB in the missing outcome data domain. Regarding the blinding of the assessors, only eight studies reported blinding of the assessors [36, 39, 41, 42, 46, 48, 51, 53]. Finally, in the domain of bias in the selection of the reported result all studies had a low RoB.

### Mean effect size and heterogeneity analysis

Meta-analyses were performed for the variables behaviour, knowledge, trunk flexion muscle endurance, trunk extension muscle endurance, hamstring flexibility and posture in the posttest and behaviour and knowledge in the follow-up. In the next paragraphs, we present the results for effect sizes comparing the treatment and control groups from baseline to the end of the intervention period (posttest).

**Fig. 2:**
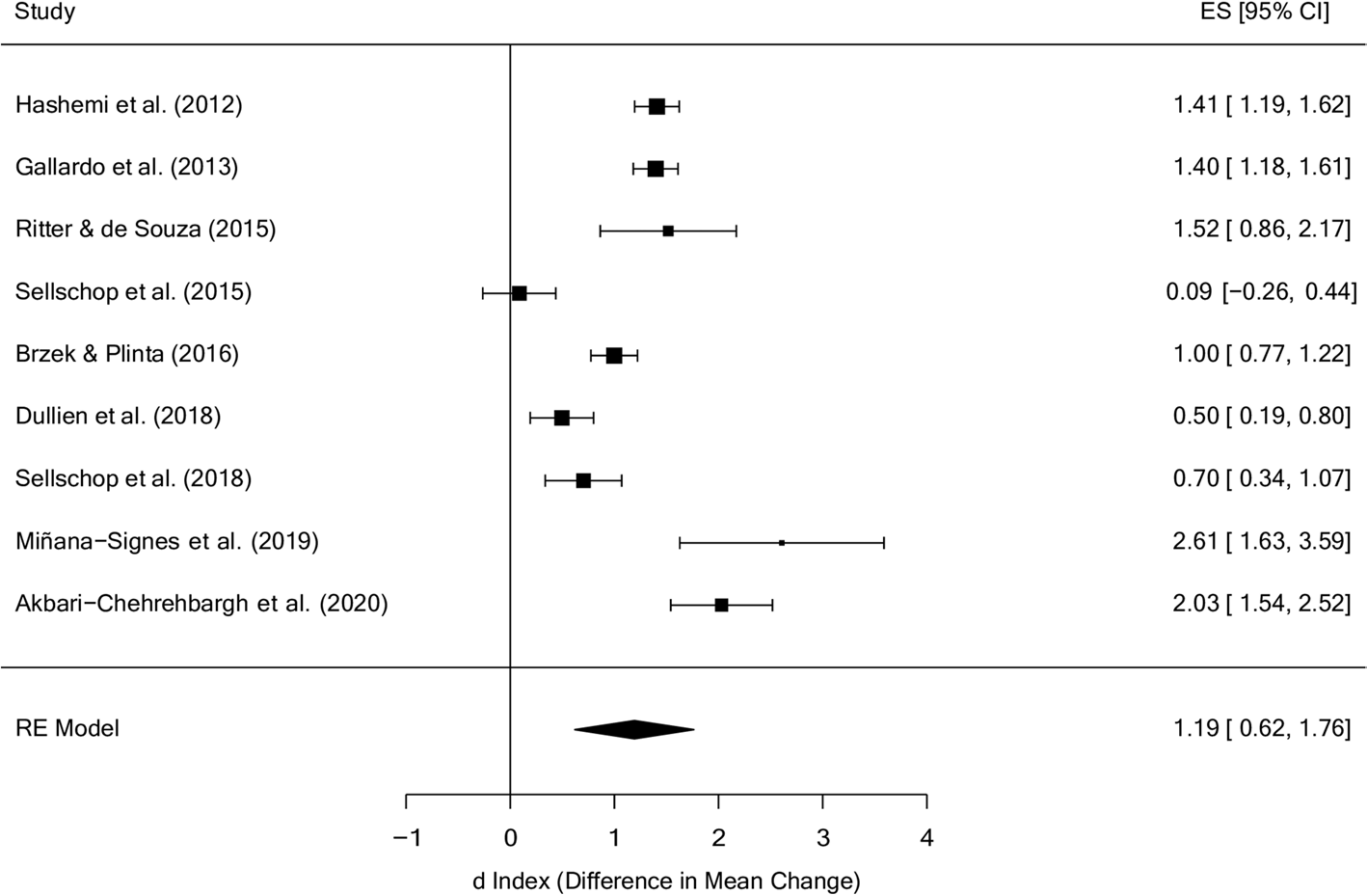
Forest plot of effect sizes for measures of behaviour in the posttest

Figure 2 presents a forest plot for the behaviour measures in the posttest, with a mean effect size of d_+_ = 1.19 (95% CI: 0.62 and 1.76), with *I*^2^ = 94.68% of the total variability due to heterogeneity. Only one study did not obtain significant differences in favor of the treatment [43], this study was also the one that carried out the least intense treatment (0.75 h per week) together with two other studies [39, 47] and with the shortest total treatment time (0.75 h, same as Sellschop et al., 2018). Furthermore, the study with the largest effect size [51] was the one that applied the most intense treatment (2.625 h per week).

**Fig. 3:**
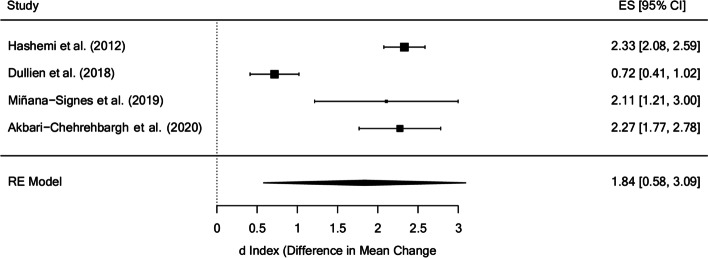
Forest plot of effect sizes for measures of knowledge in the posttest

The mean effect size of the knowledge measures in the posttest was d_+_ = 1.84 (95% CI: 0.58 and 3.09), with *I*^2^ = 93.69% of the total variability due to heterogeneity (as opposed to random sampling error). All the studies had statistically significant results in favor of the treatment, although the study that obtained the smallest effect size [46] was also the one that carried out the least intense intervention (0.31 h per week, 3.72 h in total) and the one with the longest treatment program (12 weeks). The effect sizes of the rest of the studies were very similar (Fig. 3).

**Fig. 4:**
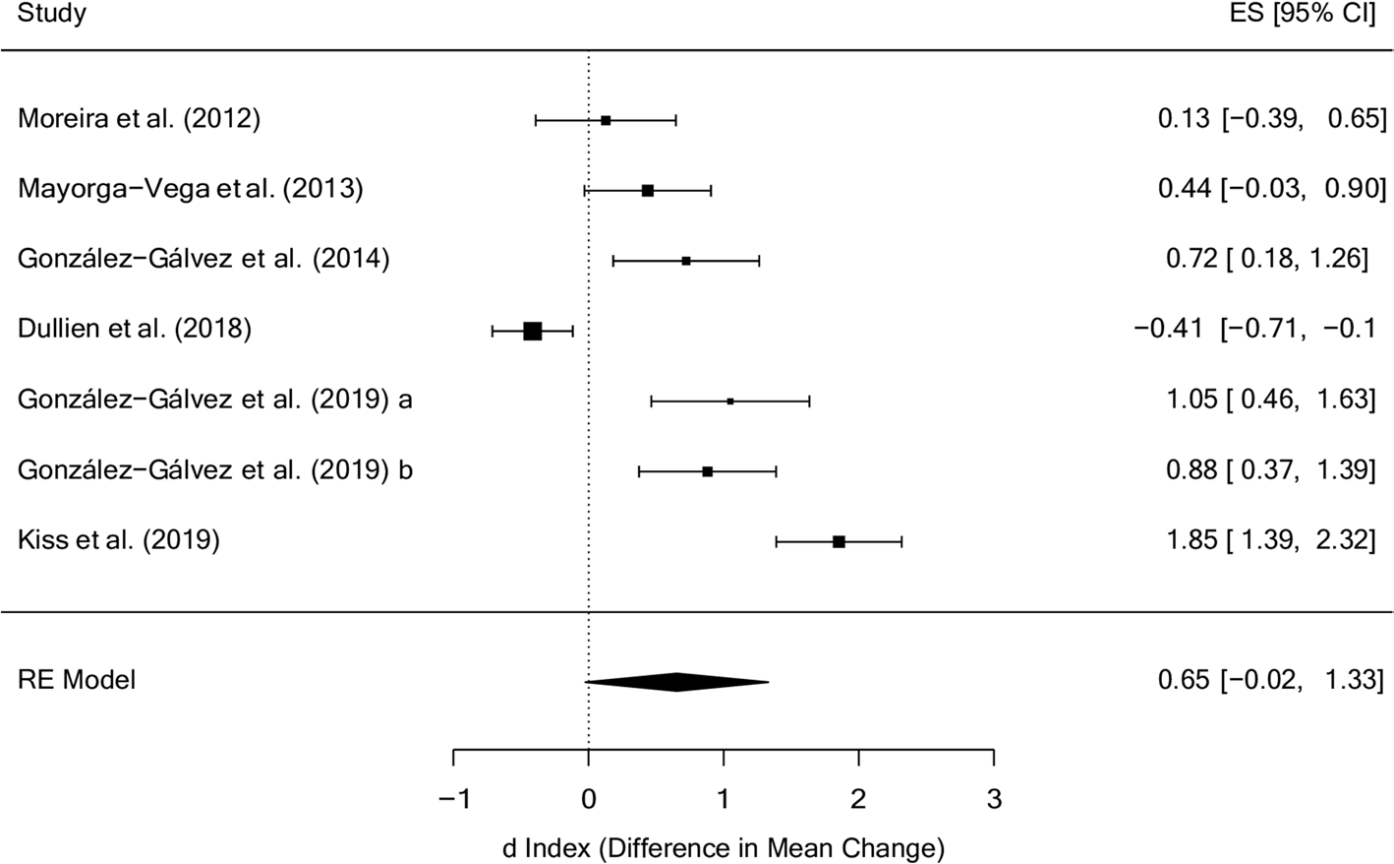
Forest plot of effect sizes for measures of trunk flexion endurance in the posttest

With regards to trunk flexion endurance in the posttest (Fig. 4) the mean effect size estimate was d_+_ = 0.65 (95% CI: -0.02 and 1.33), with I^2^ = 89.8% of the total variability attributed to true heterogeneity. The only study with statistically significant effects in favor of the control group [46] was the one with the lowest intensity (0.31 h per week) and total time of treatment (3.72 h). On the other hand, the study with the largest effect size favoring the intervention [50] was the one with the highest intensity (2.5 h per week) and total treatment time (60 weeks). It should also be mentioned that the only two studies that did not carry out progressive treatment were the ones with the smallest effect size [37, 46]. Finally, the 3 studies with the smallest effect size [37, 41, 46] were the only ones that carried out this treatment in children and the rest in adolescents.

**Fig. 5:**
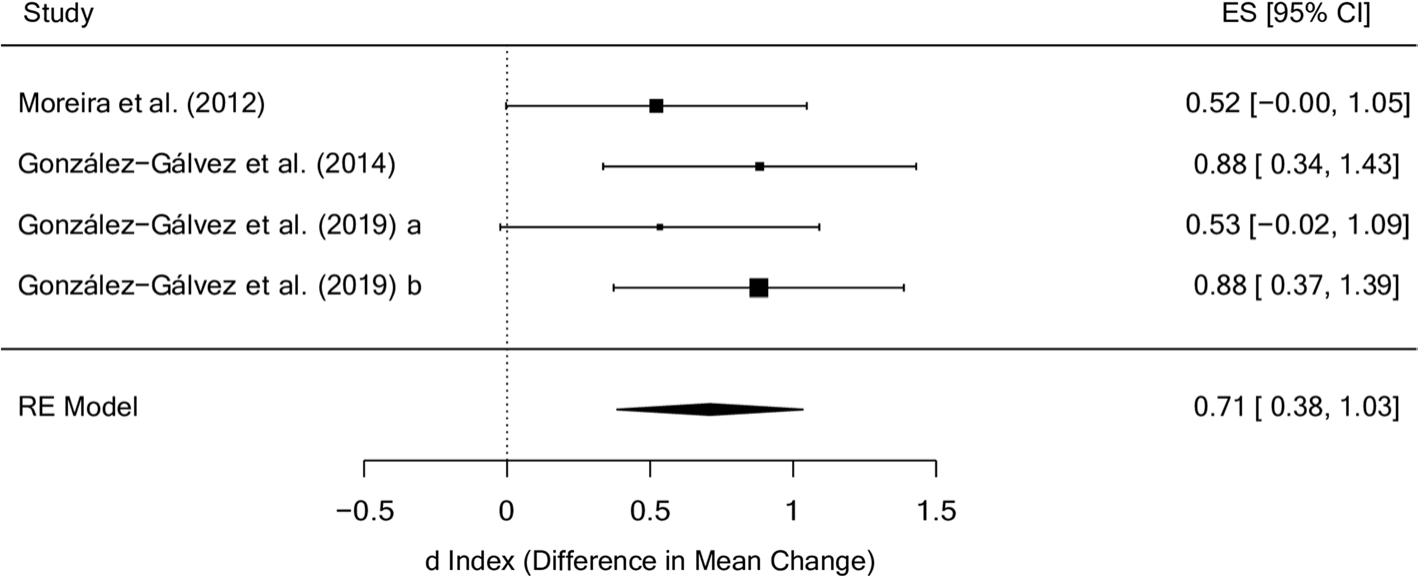
Forest plot of effect sizes for measures of trunk extension endurance in the posttest

Figure 5 presents a forest plot for the trunk extension endurance in the posttest, with a mean effect size of d_+_ = 0.71 (95% CI: 0.38 and 1.03) and no evidence of heterogeneity (I^2^ = 0%). Although two studies reported non-significant effects [37, 48], the number of weeks was the same in all studies (6 weeks) and the intensity and total time of treatment were also very similar.

**Fig. 6:**
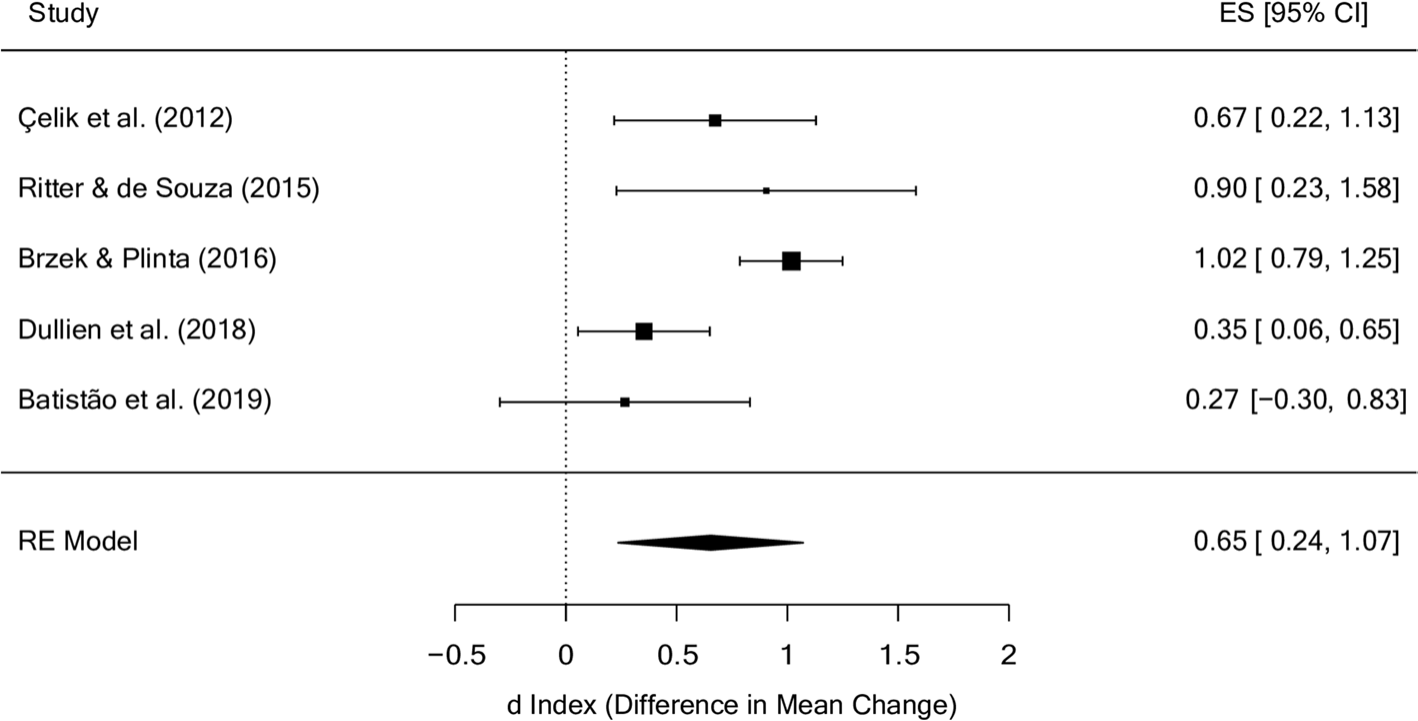
Forest plot of effect sizes for measures of posture in the posttest

The meta-analysis for posture in the posttest (Fig. 6) yielded a mean effect size of d_+_ = 0.65 (95% CI: 0.24 and 1.07), with I^2^ = 67.86%. The only study with a non-significant effect size is the only one that did not perform postural hygiene or postural correction exercises [52]. In addition, this is the study that carries out the fewest weeks of treatment (8 weeks) except for a study that does not specify the number of weeks [45].

**Fig. 7:**
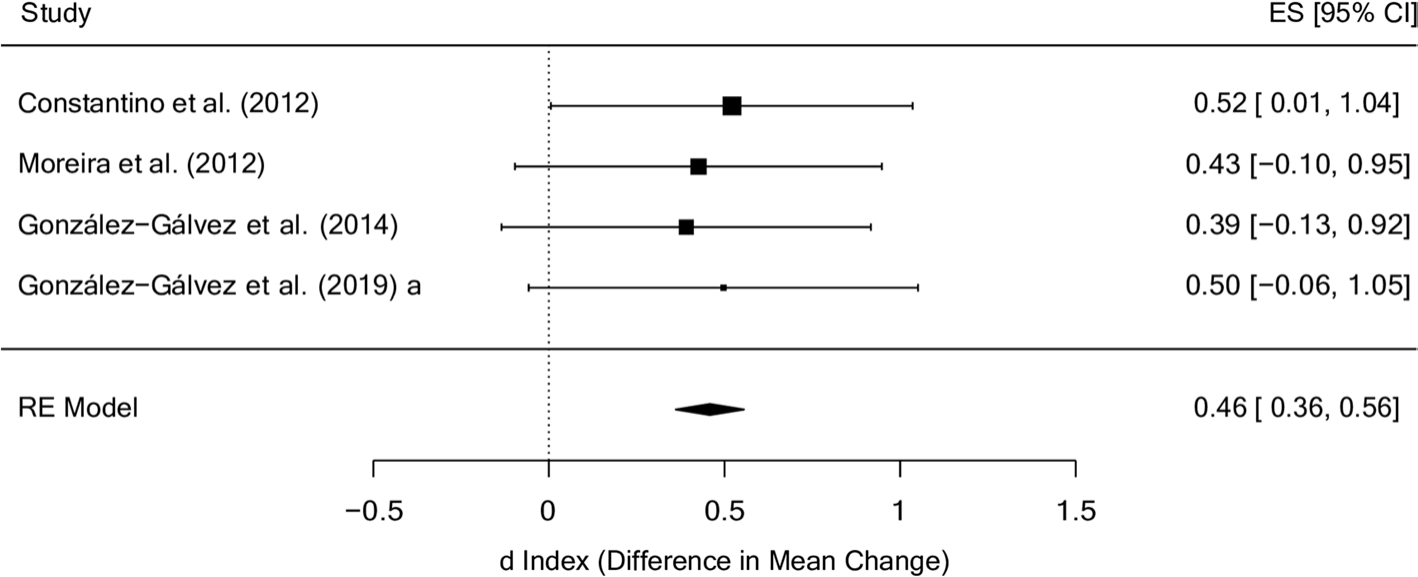
Forest plot of effect sizes for measures of hamstring flexibility in the posttest

The mean effect size estimate for hamstring flexibility in the posttest (Fig. 7) was d_+_ = 0.46 (95% CI: 0.36 and 0.56) with no evidence of heterogeneity (I^2^ = 0%). Three of the four studies reporting on this variable did not obtain statistically significant differences [37, 42, 48]. The only study that obtained statistically significant improvement [35] was the only one whose sample was made up of children, whereas the rest were made up of adolescents. Furthermore, this study was the one with the most weeks of treatment (12 weeks) and the one with the least intensity (0.23 h per week) and total treatment time (2.8 h).

**Fig. 8:**
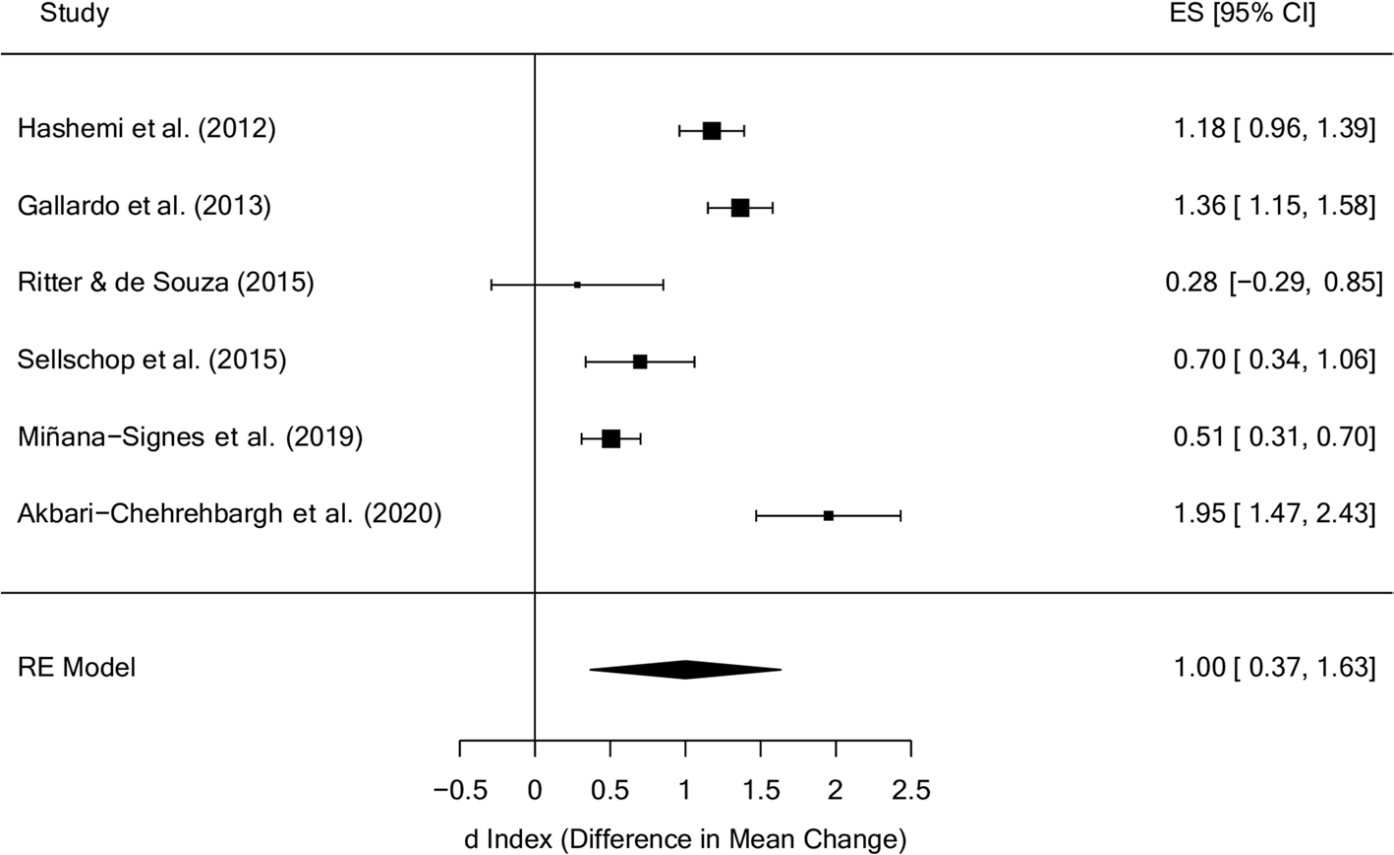
Forest plot of effect sizes for measures of behaviour in the follow-up

Regarding effect sizes from baseline to follow-up, Fig. 8 shows that overall effect size estimate for behaviour was d_+_ = 1.00 (95% CI: 0.37 and 1.63), with I^2^ = 93.91% of the total variability attributed to heterogeneity. Follow-up times ranged from 1 month to 12 months. The only study with non-significant improvements [44] is also the one with the longest follow-up (12 months), despite being the study that comprised the longest period and reported the largest treatment time.

Only two studies reported follow-up on the knowledge variable, with an overall effect size of d_+_ = 2.08 (95% CI: -0.85 and 5.02) and I^2^ = 63.08%. Follow-up for both studies was 3 months.

Other variables that could not be analyzed because they did not have a sufficient number of studies or did not have clinical relevance were: lower limb power [35, 38]; sitting time, standing time, stepping, step counts and sit-to-stand counts [40]; upper limbs muscular endurance [41, 46]; cardiovascular endurance [41]; perception [44]; lumbar motor control [50]; balance [46]; skills, self-efficacy and beliefs [53].

### Analyzing moderator variables

We examined moderating variables on the outcome variables behaviour and trunk flexion endurance. Potential moderating variables were chosen based on the clinical judgment of the authors. Due to the small number of studies of these variables, the number of moderating variables to be analyzed is limited. Some ANOVAs for the qualitative variables and simple meta-regressions for the quantitative variables were applied.

### Outcome variable: behaviour in the posttest

Tables 1 and 2 present the results of the ANOVAs and meta-regressions for behaviour. Regarding qualitative variables, the studies that were carried out in children, used theoretical and practical methods and were assessed at high RoB is high, showed larger effect sizes on average, however statistical significance was not observed in any of the moderating variables analyzed. With regards to quantitative variables, no statistical significance was observed in any of the moderating variables analyzed, however, intensity was close to statistical significance (*p* = .052), suggesting that more intense interventions might yield effects of larger magnitude (b = 0.913, 95% CI -0.015 to 1.841).

**Table 1:** Results of the weighted ANOVAs for the behaviour measures in the posttest, taking qualitative moderator variables as independent variables

Variable	*k*	d_+_	95% CI		ANOVA results
			LL	LU	
Type of treatment:					
Postural Hygiene (PH)	3	1.267	0.221	2.314	* F*(1,7) = 0.040, *p* = .847
PH + Physical exercise (PE)	6	1.157	0.374	1.940	
Age:					
Adolescent	3	0.728	-0.232	1.689	F(1,7) = 1.882, *p* = .212
Children	6	1.409	0.735	2.083	
Teaching method of PH:					
Theoretical (TT)	2	0.598	-0.540	1.736	* F*(1,7) = 1.941, *p* = .206
TT + Practical	7	1.364	0.735	1.993	
Type of behaviour:					
Schoolbag weight (SW)	5	1.118	0.199	2.036	* F*(1,7) = 0.078, *p* = .788
Healthy back habits (HBH)	4	1.266	0.412	2.120	
Risk of bias:					
High	3	1.602	0.549	2.654	* F*(1,7) = 1.216, *p* = .306
Some concerns	6	1.014	0.324	1.705	

*k *number of studies, d_+ _mean coefficient alpha, LL and LU lower and upper 95% confidence limits for d_+_, *F *Knapp-Hartung’s statistic for testing the significance of the moderator variable

**Table 2:** Results of the simple meta-regressions for the behaviour measures in the posttest, taking continuous moderator variables as predictors

Predictor variable	*k*	*b*_j_	*CI.LL*	*CI.UL*	*F*	*p*
Number of weeks of treatment	7	-0.000	-0.223	0.222	0.000	0.996
Time of treatment per week (intensity)	7	0.913	-0.015	1.841	6.400	0.052
Total time of treatment (magnitude)	8	0.068	-0.076	0.212	1.341	0.291
Total posttest sample size	9	-0.000	-0.005	0.004	0.150	0.710

*k *number of studies, *b*_j_regression coefficient of each predictor, *CI.LL *confidence interval of lower limit, *CI.UL* confidence interval of upper limit, *F *Knapp-Hartung’s statistic for testing the significance of the predictor (the degrees of freedom for this statistic are 1 for the numerator and *k* – 2 for the denominator) *p *probability level for the *F* statistic

### Outcome variable: trunk flexion endurance in the posttest

Tables 3 and 4 present the results of the ANOVAs and meta-regressions with trunk flexion endurance as the outcome variable. For qualitative variables, studies carried out in adolescents as opposed to children (*p* = .029) and those that implemented progressive training (*p* = .035) showed significantly larger effect estimates on average. Regarding quantitative variables, the intensity (b = 0.869, 95% CI 0.225 to 1.513; *p* = .018) and magnitude (b = 0.031, 0.004 to 0.057; *p* = .030) showed statistically significant direct relationships with effect size.

**Table 3:** Results of the weighted ANOVAs for the trunk flexion endurance measures in the posttest, taking qualitative moderator variables as independent variables

Variable	*k*	d_+_	95% CI		ANOVA results
			LL	LU	
Type of treatment:					
Physical exercise (PE)	4	0.536	-0.471	1.543	* F*(1,5) = 0.211, *p* = .665
PE + Physical activity (PA)	3	0.811	-0.350	1.972	
Age:					
Adolescent	4	1.140	0.505	1.774	F(1,5) = 9.239, *p* = .029
Children	3	0.024	-0.675	0.722	
Progression:					
Yes	5	0.992	0.396	2.654	* F*(1,5) = 7.642, *p* = .035
No	2	-0.169	-1.069	1.588	
Risk of bias:					
High	1	0.722	-1.353	2.797	* F(1,5) = 0.008, p = .932*
Some concerns	6	0.644	-0.193	1.480	

*k *number of studies, d_+_mean coefficient alpha, *LL* and *LU *lower and upper 95% confidence limits for d_+_. *F * Knapp-Hartung’s statistic for testing the significance of the moderator variable

**Table 4:** Results of the simple meta-regressions for the trunk flexion endurance measures in the posttest, taking continuous moderator variables as predictors

Predictor variable	*k*	*b*_j_	*CI.LL*	*CI.UL*	*F*	*p*
Number of weeks of treatment	7	0.054	-0.055	0.163	1.612	0.260
Time of treatment per week (intensity)	7	0.869	0.225	1.513	12.026	0.018
Total time of treatment (magnitude)	7	0.031	0.004	0.057	8.958	0.030
Total posttest sample size	7	-0.006	-0.024	0.011	0.897	0.387

*k *number of studies, *b*_j_regression coefficient of each predictor, *CI.LL* confidence interval of lower limit, *CI.UL* confidence interval of upper limit, *F*Knapp-Hartung’s statistic for testing the significance of the predictor (the degrees of freedom for this statistic are 1 for the numerator and *k* – 2 for the denominator), *p* probability level for the *F* statistic

### Publication bias

Due to the small number of studies for each variable, publication bias was only examined using meta regression models with n at posttest as a covariate. The behaviour variable resulted in a b_j_= -0.000 (*p* = .710) and the trunk flexion endurance variable resulted in a b_j_= -0.006 (*p* = .387). A negative sign is compatible with the hypothesis of publication bias, and the non-significant p-value suggests no publication bias concerns, but could also be due to the lack of statistical power with such a low number of studies.

### Certainty of evidence

The GRADE system was applied to each variable ranging from very low to moderate certainty of evidence. For more information, see Additional file 5.

## Discussion

This systematic review and meta-analysis examined the effectiveness of physiotherapy interventions for back care in children and adolescents, updating the state of the art in this field. Clinical trials after 2012 were collected in order to compare the results with the latest published meta-analysis [21].

In the studies included in this meta-analysis, clinical reasoning is supported by knowledge from the literature, and the methods for testing the hypotheses of each of them are adequate.

The variables analyzed in this meta-analysis are currently still being studied in clinical trials. Recent studies, that were not included in this meta-analysis because they did not meet the inclusion criteria, investigated physiotherapy to improve back care by increasing behaviour [54], knowledge and posture [55].

The results indicate that physiotherapy effectively improved behaviour in the posttest (d_+_ = 1.19) and follow-up (d_+_ = 1.00). These effect sizes are, respectively, similar (d_+_ = 1.33) and lower (d_+_ = 1.80) than the previous meta-analysis that examined this variable [21]. These findings are reinforced by a recent systematic review which argued that an intervention in children and adolescents can improve behaviour related to back care and the research on back health is scarce in this population [20].

The results indicate that physiotherapy significantly improved knowledge with an effect size of d_+_ = 1.19, similar to the previous meta-analysis (d_+_ = 1.29) [21]. Since we only included four studies for this variable, an analysis of the moderator variables was not considered. The effect size we estimated at follow-up with a d_+_ = 2.08 was larger than in the previous meta-analysis (d_+_ = 0.76) [21]. Previous systematic reviews argue that the teaching of knowledge in children and adolescents is important to improve back care [19, 20]. Some of the RCTs included in those systematic reviews were also included in the current meta-analysis.

Concerning to the trunk flexion endurance, this was the only variable yielding non-significant results with an effect size of d_+_ = 0.65. Such lack of statistical significance may be due to the small number of studies.

The results indicate that physiotherapy also improved trunk extension endurance with an effect size of d_+_ = 0.71. Although the four studies reporting on this variable were very similar, a large difference was observed between two studies with statistically significant results [42, 49] and two studies with non-significant findings [37, 48]. Due to the small number of studies, an analysis of the moderating variables was not carried out.

Regarding hamstring flexibility, a significant effect size of d_+_ = 0.46 was obtained. At least six weeks of treatment may be required to obtain improvements but at least twelve weeks are necessary for this improvement to be statistically significant. This statement aligns with findings from previous systematic reviews [16, 57]. Some studies that assessed hamstring flexibility were not included because the treatment was not aimed at improving back care.

In the posture variable, a significant effect size of d_+_ = 0.71 was obtained. To achieve an improvement in posture, postural hygiene or postural correction exercises are essential. Posture assessment tools varied widely across studies, including instruments such as the New York Posture Rating [34] or the Postural Evaluation Software [52]. Previous systematic reviews also highlight the use of different assessment tools across studies [18].

Concerning to moderator variables in behaviour, the previous meta-analysis reported that the type of postural hygiene and the postural hygiene teaching method are moderator variables that influence the effect size [21]. However, in the current study we could not calculate the influence of the type of postural hygiene because all the studies included used the same strategy (knowledge acquisition + posture habits training). This may be because after 2012 clinical trials have taken into account the results of the last published meta-analysis [21] which showed that this is the best combination. Regarding the postural hygiene teaching method, we did not find significant differences, although the studies that carried out a theoretical and practical treatment obtained better results than those that only used theoretical treatment. Overall, interventions yielded slightly more effective results for children than for adolescents. Due to the heterogeneity of the tools to assess the results, the type of behaviour (backpack weight vs. healthy back habits) was analyzed as a moderator variable, but no differences were found between them. Studies with a high risk of bias obtained somewhat larger effect sizes than studies with some bias concerns, but this difference did not reach statistical significance. Moreover, the number of weeks of treatment was not a significant moderator variable as in our previous study [21]. The intensity of the treatment as a moderator variable was close to being significant, as in our previous study [21], however, the magnitude of the treatment was significant in the previous, but not in the current study. Finally, the total posttest sample size was not a significant moderating variable.

In relation to moderator variables in trunk flexion endurance, studies that involved physical exercise and physical activity had better results than those that involved physical exercise alone with no significant differences, this may be due to the fact that physical activity has worked other muscle group than those worked by physical exercise or has reinforced those already worked by exercise. The studies carried out in adolescents obtained better results than those carried out in children (*p* = .029), which is in agreement with previous meta-analysis [58], but opposite to another systematic review that found no differences regarding strength gain [59]. Studies that carried out the treatment progressively yielded a significantly larger improvement compared to those that did not (*p* = .035), which supports claims from other authors who defended the need for the treatment to be progressive in order to obtain better results [15]. The study assessed at high risk of bias showed effect sizes of larger magnitude than studies with some bias concerns. The number of weeks was not a significant moderating variable, although another study recommends that treatment should last longer than 8 weeks [15]. The intensity (*p* = .018) and magnitude (*p* = .030) were variables that influenced the effect size, as stated in another study [15]. Finally, the total posttest sample size was not a significant moderating variable.

This study will allow clinicians to treat patients with the most effective treatments based on current evidence to achieve better outcomes, researchers will be able to develop new research projects that improve the quality of evidence, and patients will be able to apply the knowledge obtained from this study to improve their health.

### Strengths

To our knowledge, this is the only meta-analysis since 2012 that evaluated the effectiveness of physiotherapy interventions for back care in children and adolescents. Other strengths include use of the Cochrane Risk of Bias 2 guideline and the GRADE system for recommendations. Two reviewers independently extracted key study data, evaluated RoB and applied the GRADE framework to the findings of each meta-analysis. Furthermore, a wide range of outcomes was considered.

### Limitations

There is substantial variation across studies in terms of interventions, and comparators make it difficult to pinpoint the exact source of this diversity. In addition, there were few studies within each meta-analysis to explore sources of heterogeneity. Another limitation relates to the high RoB in the studies.

### Implications for future research

Firstly, studies comparing various treatment groups in terms of intensity, types of exercise, and progression are needed. Secondly, studies must ensure that participants are randomized and those evaluators are blinded. Finally, future studies should evaluate the results with the same tool, which requires validated tools in this population.

## Conclusions

The most effective preventive physiotherapy interventions to improve back care in children and adolescents and prevent non-specific low back pain were physical exercise, postural hygiene and physical activity. These treatments achieved statistically significant improvements in back care knowledge, back care behaviour, posture, trunk extension endurance and hamstring flexibility. Based on GRADE methodology, we found that the evidence ranged from very low to moderate quality.

## Supplementary Information

**Additional file 1.**

**Additional file 2.**

**Additional file 3.**

**Additional file 4.**

**Additional file 5.**

**Additional file 6.**

## Data Availability

All data generated or analyzed during this study are included in the Additional file 6.

## Abbreviations

LBP: Low back pain
NSLBP: Non-specific low back pain
RCTs: Randomized controlled trials
WoS: Web of Science
BMJ: British Medical Journal
ICC: Intraclass correlation coefficient
RoB: Risk of bias

## References

[1] Minghelli B (2017). Low Back Pain in Childhood and Adolescent Phase: Consequences, Prevalence and Risk Factors – A Revision. J Spine.

[2] Hwang J, Louie PK, Phillips FM, An HS, Samartzis D (2019). Low back pain in children: a rising concern. Eur Spine J.

[3] Calvo-Muñoz I, Kovacs FM, Roqué M, Gago Fernández I, Seco Calvo J (2018). Risk Factors for Low Back Pain in Childhood and Adolescence: A Systematic Review. Clin J Pain.

[4] O’Sullivan PB, Smith AJ, Beales DJ, Straker LM (2011). Association of biopsychosocial factors with degree of slump in sitting posture and self-report of back pain in adolescents: a cross-sectional study. Phys Ther.

[5] Batley S, Aartun E, Boyle E, Hartvigsen J, Stern PJ, Hestbæk L (2019). The association between psychological and social factors and spinal pain in adolescents. Eur J Pediatr.

[6] O’Sullivan K, O’Keeffe M, Forster BB, Qamar SR, van der Westhuizen A, O’Sullivan PB (2019). Managing low back pain in active adolescents. Best Pract Res Clin Rheumatol.

[7] Miñana-Signes V, Monfort-Pañego M (2016). Knowledge on health and back care education related to physical activity and exercise in adolescents. Eur Spine J.

[8] Aparicio-Sarmiento A, Rodríguez-Ferrán O, Martínez-Romero MT, Cejudo A, Santonja F, Sainz de Baranda P (2019). Back Pain and Knowledge of Back Care Related to Physical Activity in 12 to 17 Year Old Adolescents from the Region of Murcia (Spain): ISQUIOS Programme. Sustainability.

[9] Lobo ME, Kanagaraj R, Jidesh V (2013). An insight into adolescents’ knowledge and attitudes on low back pain and its occurrence. Int J Ther Rehabil.

[10] Alsiddiky A, Alatassi R, Alsaadouni FN, Bakerman K, Awwad W, Alenazi A (2019). Assessment of perceptions, knowledge, and attitudes of parents regarding children’s schoolbags and related musculoskeletal health. J Orthop Surg.

[11] O’Sullivan P, Smith A, Beales D, Straker L (2017). Understanding Adolescent Low Back Pain From a Multidimensional Perspective: Implications for Management. J Orthop Sports Phys Ther.

[12] Bettany-Saltikov J, Kandasamy G, Schaik PV, McSherry R, Hogg J, Whittaker V (2019). School-based education programmes for improving knowledge of back health, ergonomics and postural behaviour of school children aged 4–18: A systematic review. Campbell Syst Rev.

[13] Bortone I, Agnello N, Argentiero A, Denetto V, Neglia C, Palestra G, et al. The PoSE Project: the Potential of Technological Learning for Postural Education in Schoolchildren. EAI Endorsed Trans E Learn. 2015;2(6):1–7.

[14] Allen BA, Hannon JC, Burns RD, Williams SM (2014). Effect of a core conditioning intervention on tests of trunk muscular endurance in school-aged children. J Strength Cond Res.

[15] Lloyd RS, Faigenbaum AD, Stone MH, Oliver JL, Jeffreys I, Moody JA (2014). Position statement on youth resistance training: the 2014 International Consensus. Br J Sports Med.

[16] Becerra-Fernández CA, Mayorga-Vega D, Merino-Marbán R (2020). Effect of Physical Education-based stretching programs on hamstring extensibility in high school students: A systematic review. Cult Cienc Deporte.

[17] Alibegović A, Mačak Hadžiomerović A, Pašalić A, Domljan D (2020). School Furniture Ergonomics in Prevention of Pupils’ Poor Sitting Posture. Drv Ind Znan Časopis Za Pitanja Drv Tehnol.

[18] Dugan JE (2018). Teaching the body: a systematic review of posture interventions in primary schools. Educ Rev.

[19] Chacón-Borrego F, Jiménez JLU, García JJLG, Ruz RP, González M del MC. Educación e higiene postural en el ámbito de la Educación Física. Papel del maestro en la prevención de lesiones. Revisión sistemática. Retos. 2018;(34):8–13.

[20] Miñana-Signes V, Monfort-Pañego M, Valiente J. Teaching Back Health in the School Setting: A Systematic Review of Randomized Controlled Trials. Int J Environ Res Public Health. 2021;18(3):979. 10.3390/ijerph18030979 PMC7908500 33499403

[21] Calvo-Muñoz I, Gómez-Conesa A, Sánchez-Meca J (2012). Preventive physiotherapy interventions for back care in children and adolescents: a meta-analysis. BMC Musculoskelet Disord.

[22] Page MJ, McKenzie JE, Bossuyt PM, Boutron I, Hoffmann TC, Mulrow CD (2021). Updating guidance for reporting systematic reviews: development of the PRISMA 2020 statement. J Clin Epidemiol.

[23] Lipsey MW (2019). Identifying interesting variables and analysis opportunities. The handbook of research synthesis and meta-analysis.

[24] Orwin RG, Vevea JL (2009). Evaluating coding decisions. The handbook of research synthesis and meta-analysis.

[25] Sterne JAC, Savović J, Page MJ, Elbers RG, Blencowe NS, Boutron I (2019). RoB 2: a revised tool for assessing risk of bias in randomised trials. BMJ.

[26] Borenstein M, Hedges LV, Higgins JPT, Rothstein HR. Introduction to meta-analysis. Chichester: Wiley; 2009.

[27] Rubio-Aparicio M, Marín-Martínez F, Sánchez-Meca J, López-López JA (2018). A methodological review of meta-analyses of the effectiveness of clinical psychology treatments. Behav Res Methods.

[28] Morris SB (2008). Estimating Effect Sizes From Pretest-Posttest-Control Group Designs. Organ Res Methods.

[29] Cohen J (1988). Statistical power analysis for the behavioral sciences.

[30] Hartung J (1999). An Alternative Method for Meta-Analysis. Biom J.

[31] Knapp G, Hartung J (2003). Improved tests for a random effects meta-regression with a single covariate. Stat Med.

[32] R Core Team. R: A language and environment for statistical computing. R Foundation for Statistical Computing, Vienna, Austria. URL https://www.R-project.org/. 2022.

[33] Viechtbauer W (2010). Conducting Meta-Analyses in R with the metafor Package. J Stat Softw.

[34] Kayapinar FC, Mengutay S, Uzun S (2012). The Investigation Effects of Sample Pilot Study Program on Postur of Preschool Children. Procedia - Soc Behav Sci.

[35] Coledam DHC, de Arruda GA, de Oliveira AR (2012). Effects of an exercise program on children’s flexibility and vertical jump performance. Mot Rev Educ Física.

[36] Habybabady RH, Ansari-Moghaddam A, Mirzaei R, Mohammadi M, Rakhshani M, Khammar A (2012). Efficacy and impact of back care education on knowledge and behaviour of elementary schoolchildren. JPMA J Pak Med Assoc.

[37] Moreira RFC, Akagi FH, Wun PYL, Moriguchi CS, Sato TO (2012). Effects of a school based exercise program on children’s resistance and flexibility. Work Read Mass.

[38] Müller J, Otto C, Stoll J, Müller S, Mayer F. Effects of six-months trunk stability exercises on low back pain prevalence in young athletes. In San Francisco, United States; 2012.

[39] Gallardo Vidal MI, Rodríguez Barrientos R, Borda Olivas A (2013). Evaluación de la efectividad de una intervención educativa para disminuir el peso de la mochila escolar en los alumnos de 3.° y 4.° de educación primaria. Fisioterapia.

[40] Hinckson EA, Aminian S, Ikeda E, Stewart T, Oliver M, Duncan S (2013). Acceptability of standing workstations in elementary schools: a pilot study. Prev Med.

[41] Mayorga-Vega D, Viciana J, Cocca A (2013). Effects of a Circuit Training Program on Muscular and Cardiovascular Endurance and their Maintenance in Schoolchildren. J Hum Kinet.

[42] González-Gálvez N, Poyatos MC, Pardo PJM, Feito Y (2014). The Effect of Pilates Method in Scholar’s Trunk Strength and Hamstring Flexibility: Gender Differences. Int J Sport Health Sci.

[43] Sellschop I, Myezwa H, Mudzi W, Mbambo-Kekana N (2015). The effect of a computer-related ergonomic intervention program on learners in a school environment. Work Read Mass.

[44] Ritter AL, de Souza JL, Ritter AL, de Souza JL (2015). Postural education program for elementary school: a one-year follow-up study. Mot Rev Educ Física.

[45] Brzek A, Plinta R (2016). Exemplification of Movement Patterns and Their Influence on Body Posture in Younger School-Age Children on the Basis of an Authorial Program ‘I Take Care of My Spine’. Medicine (Baltimore).

[46] Dullien S, Grifka J, Jansen P (2018). Cluster-randomized, controlled evaluation of a teacher led multi factorial school based back education program for 10 to 12-year old children. BMC Pediatr.

[47] Sellschop IV, Myezwa H, Mudzi W, Musenge E (2018). Ergonomic behaviour of learners in a digitally driven school environment: Modification using an ergonomic intervention programme. South Afr J Physiother.

[48] González-Gálvez N, Marcos-Pardo PJ, Carrasco-Poyatos M (2019). Functional improvements after a pilates program in adolescents with a history of back pain: A randomised controlled trial. Complement Ther Clin Pract.

[49] González-Gálvez N, Poyatos MC, Marcos-Pardo PJ, Feito Y, Vale RG, de González-Gálvez S (2019). Pilates training induces changes in the trunk musculature of adolescents. Rev Bras Med Esporte.

[50] Kiss G, Kovácsné VB, Tóth ÁL, Jeges S, Makai A, Szilágyi B (2019). Efficiency examination of a 6-month trunk prevention program among recruitment kayak-canoe athletes: A randomized control trial. J Back Musculoskelet Rehabil.

[51] Miñana-Signes V, Monfort-Pañego M, Rosaleny-Maiques S (2019). Improvement of knowledge and postural habits after an educational intervention program in school students. J Hum Sport Exerc.

[52] Batistão MV, Carnaz L, Moreira R de FC, Sato T de O, Carnaz L, Moreira R de FC, et al. Effects of a muscular stretching and strengthening school-based exercise program on posture, trunk mobility, and musculoskeletal pain among elementary schoolchildren - a randomized controlled trial. Fisioter Em Mov. 2019;32:1–9.

[53] Akbari-Chehrehbargh Z, Tavafian SS, Montazeri A (2020). Effectiveness of a theory-based back care intervention on spine-related behavior among pupils: a school-based randomised controlled trial (T-Bak study). BMC Public Health.

[54] Rodríguez-Oviedo P, Santiago-Pérez MI, Pérez-Ríos M, Gómez-Fernández D, Fernández-Alonso A, Carreira-Núñez I (2018). Backpack weight and back pain reduction: effect of an intervention in adolescents. Pediatr Res.

[55] Szilágyi B, Makai A, Tardi P, Kukla A, Járomi M. Evaluation and development of knowledge of spinal function and posture with back school program among primary school children. 2019;3:39–53.

[56] Hill JJ, Keating JL (2015). Daily exercises and education for preventing low back pain in children: cluster randomized controlled trial. Phys Ther.

[57] Becerra Fernandez C-A, Merino-Marban R (2015). Efficacy of hamstring stretching programs in schoolchildren. A systematic review. Timisoara Phys Educ Rehabil J.

[58] Behringer M, Vom Heede A, Yue Z, Mester J (2010). Effects of resistance training in children and adolescents: a meta-analysis. Pediatrics.

[59] Peitz M, Behringer M, Granacher U (2018). A systematic review on the effects of resistance and plyometric training on physical fitness in youth- What do comparative studies tell us?. PloS One.

