# Supplementary material for: Effectiveness of physiotherapy interventions for back care and the prevention of non-specific low back pain in children and adolescents: a systematic review and meta-analysis
Source: BMC Musculoskelet Disord. 2022 Apr 2;23:314. doi: 10.1186/s12891-022-05270-4 (PMC8976404; doi:10.1186/s12891-022-05270-4)
Supplement: Supplementary file 1 — Additional file 1. [file 12891_2022_5270_MOESM1_ESM.docx]

**Search strategy**

Title: Effectiveness of physiotherapy interventions for back care and the prevention of non-specific low back pain in children and adolescents: a systematic review and meta-analysis

| **Database** | **Search strategy** | **Number of studies included** |
| --- | --- | --- |
| Medline | (adolescent* OR child* OR young* OR school*) AND (“back pain” OR “low back pain” OR “back complaint” OR “back care”) AND (prevention OR education OR “postural hygiene” OR “physical education” OR “back education” OR “posture education” OR “back function” OR physiotherapy OR backpack OR ergonomics OR “physical therapy” OR “exercise therapy” OR promotion OR knowledge OR behaviour OR “cognitive behavioral therapy”)  Filters: publication date from 2012/05/01; age 0-18 | 1 |
| Web of Science | (adolescent* OR child* OR young* OR school*) AND (“back pain” OR “low back pain” OR “back complaint” OR “back care”) AND (prevention OR education OR “postural hygiene” OR “physical education” OR “back education” OR “posture education” OR “back function” OR physiotherapy OR backpack OR ergonomics OR “physical therapy” OR “exercise therapy” OR promotion OR knowledge OR behaviour OR “cognitive behavioral therapy”)  Filters: publication date from 2012; Title/abstract | 16 |
| Cochrane Library | (adolescent* OR child* OR young* OR school*) AND (“back pain” OR “low back pain” OR “back complaint” OR “back care”) AND (prevention OR education OR “postural hygiene” OR “physical education” OR “back education” OR “posture education” OR “back function” OR physiotherapy OR backpack OR ergonomics OR “physical therapy” OR “exercise therapy” OR promotion OR knowledge OR behaviour OR “cognitive behavioral therapy”)  Filters: publication date from 2012/05/01 | 0 |
| PEDro | Adolescent* “low back pain”  Filters: publication date from 2012/05/01; Match all search items (AND) | 0 |
| PsycInfo | (adolescent* OR child* OR young* OR school*) AND (“back pain” OR “low back pain” OR “back complaint” OR “back care”) AND (prevention OR education OR “postural hygiene” OR “physical education” OR “back education” OR “posture education” OR “back function” OR physiotherapy OR backpack OR ergonomics OR “physical therapy” OR “exercise therapy” OR promotion OR knowledge OR behaviour OR “cognitive behavioral therapy”)  Filters: publication date from 2012; Chilhood (birth-12 years), Adolescent (13-17 years) | 0 |
| LILACS | Adolescent* “low back pain”  Filters: publication date from 2012 to 2020 | 0 |
| IBECS | Adolescent low back pain  Filters: match all the search items (AND) | 0 |

*Citation search: 3 studies included*
