# Supplementary material for: Effectiveness of physiotherapy interventions for back care and the prevention of non-specific low back pain in children and adolescents: a systematic review and meta-analysis
Source: BMC Musculoskelet Disord. 2022 Apr 2;23:314. doi: 10.1186/s12891-022-05270-4 (PMC8976404; doi:10.1186/s12891-022-05270-4)
Supplement: Supplementary file 2 — Additional file 2. [file 12891_2022_5270_MOESM2_ESM.docx]

The following treatment features were coded: (a) the type of preventive physiotherapy treatment (postural hygiene, physiotherapy exercise, physical activity, others); (b) the acquisition mode of postural hygiene (acquisition of knowledge, posture training habits, body awareness training, others); (c) the teaching method of postural hygiene (theoretical, practical); (d) the type of physiotherapy exercise (stretching, strengthening, pelvic tilt exercises, breathing, posture correction, balance exercises, others, It was carried out by a specific method); (e) the type of physical activity (sports, games, others); (f) the duration of the treatment (in weeks); (g) the intensity of the treatment (number of weekly hours of treatment received by each subject); (h) the magnitude of the treatment (total number of hours received by each subject); (i) the existence of an established number of sessions; (j) the homogeneity of the treatment (whether all patients received the treatment in the same conditions); (k) the inclusion of homework; (l) the inclusion of a follow-up program; (m) the use of external agents to the therapeutic group (subjects that are not part of the therapy group, who are not professionals, but who have an influence, being able to support the subjects in attaining their therapy goals); (n) the presence of family members who act as co-therapists that continue or carry out preventive treatment at home); (o) the presence of teachers who act as co-therapists that continue or carry out preventive treatment at home; (p) the mode of application of the intervention (direct, indirect or mixed); (q) the mode of training (group, individual or mixed); (r) the use of informed consent. Regarding the characteristics of the therapists the following variables were coded: (s) the number of therapists; (t) whether or not the authors agree with the therapists; (u) the training of the therapist (physiotherapist, other); (v) the experience of the therapists (large, medium, low, mixed), and (w) the gender of therapists (men, women, mixed).

Only two contextual characteristics were coded: (a) the country and (b) the place where the intervention was carried out (university, clinic, health center/day center, hospital, school, sports center, mixed).

The participant characteristics coded in the samples of each study were: (a) the mean age of the subjects and the standard deviation (SD) (in years); (b) the gender of the sample (percentage of males); (c) the physical activity level of subjects during the intervention (low, moderate, regular), and (d) whether or not they had undertaken previous treatments, (e) the percentage of participants with low back pain, and (f) the percentage of participants with spinal deformity.

The following methodological characteristics were coded: (a) how the subjects were allocated to the treatments (randomly vs. nonrandomly); (b) the type of control group (nonactive vs. active); (c) the largest follow-up in the study (in months); (d) the sample size; (e) the attrition in the posttest; (f) the attrition in the follow-up; (g) the attrition difference in the posttest and; (h) the attrition difference in the follow-up.

Finally, the extrinsic characteristics coded were: (a) the year of the study; (b) the profession of the first author (physiotherapist, ergonomist, teacher, physician, other), (c) the publication of the study (published vs. un published) and, (d) the publication source (paper, book chapter, book or monograph, unpublished manuscript, congress communication, technical report, doctoral thesis, and others).

Definitions:

- Postural hygiene: refers to interventions in which knowledge such as anatomy, biomechanics, risk factors, promotion of physical activity, healthy postural habits, breathing, etc. are acquired. Also the practice of correct postures and activities and body awareness training, which can be theoretical, practical or both.
- Exercise: all therapeutic training based on stretching, strengthening, pelvic scales, breathing exercises, postural correction, balance, etc.
- Physical activity: therapeutic physical conditioning based on regulated sports or games
- Knowledge: information about back care.
- Behaviour: ways of acting that influence in back care.
- Posture: The way in which the body or a part of the body of patients is positioned in relation to their back care.
- Trunk flexion endurance: endurance performed by the muscles of the front part of the abdomen responsible for the anterior flexion of the trunk.
- Trunk extension endurance: endurance performed by the muscles of the back of the trunk responsible for trunk extension.
- Hamstrings flexibility: elongation capacity of the back thigh muscles.
