# Supplementary material for: Effectiveness of physiotherapy interventions for back care and the prevention of non-specific low back pain in children and adolescents: a systematic review and meta-analysis
Source: BMC Musculoskelet Disord. 2022 Apr 2;23:314. doi: 10.1186/s12891-022-05270-4 (PMC8976404; doi:10.1186/s12891-022-05270-4)
Supplement: Supplementary file 4 — Additional file 4. [file 12891_2022_5270_MOESM4_ESM.docx]

Supplementary table 1. Characteristics of the studies included

| **Study** | **Design & Country** | **Participants** | **Intervention time** | **Experimental Intervention** | **Intervention descriptions** | **Outcomes** | **Risk of bias** |
| --- | --- | --- | --- | --- | --- | --- | --- |
| Kayapinar et al., 2012 | Quasi-experimental, Turkey | 128 subjects  Age: 5-7  E = 69  C = 59 | 12 weeks, 6.41 hours per week, 77 total hours | Exercise + PA | E: Posture exercises + games of accordance with ages group  C: Control | The experimental group obtained statistically significant higher scores than the control group for posture in the posttest | High |
| Constantino et al., 2012 | Quasi-experimental, Brazil | 62 subjects  Age: 9.5  E = 31  C = 31 | 12 weeks, 0.23 hours per week, 2.8 total hours | Exercise | E: Active static stretching + skipping  C: Individual and collective sports through games | The experimental group obtained statistically significant higher scores than the control group for the hamstrings flexibility and lower limb power in the posttest | High |
| Moreira et al., 2012 | RCT, Brazil | 58 subjects  Age: 12  E = 28  C = 30 | 6 weeks, 2 hours per week, 12 total hours | Exercise + PA | E: Running games + stretching + curl-up, curl-up oblique, bridge, birddog, bridge from the knees, back extension and cat-camel  C: Control | The experimental group obtained scores not statistically significant higher than the control group for the trunk flexion endurance, trunk extension endurance, and hamstring flexibility in the posttest | Some concerns |
| Müller et al., 2012 | Quasi-experimental, Germany | 140 subjects  Age: 13  E = 43  C = 97 | 24 weeks, 0.75 hours per week, 18 total hours | Exercise | E: Side-stabilization, side jumps and two-legged jumps + pelvis lift  C: usual training (no specific) | The experimental group obtained not statistically significant higher scores than the control group for the lower limb power in the posttest | High |
| Habybabady et al., 2012 | RCT, Iran | 404 subjects  Age: 10-11  E = 203  C = 201 | 4 total hours | PH | E: Anatomy and structure of spine, ergonomic about backpack + sitting posture and lying, body posture while lifting, push and pulling  C: Control | The experimental group obtained statistically significant higher scores than the control group for the behaviour and knowledge in the posttest and follow-up | Some concerns |
| Gallardo et al., 2013 | RCT, Spain | 358 subjects  Age: 8.7 (8-10)  E = 271  C = 87 | 3 weeks, 0.75 hours per week, 18 total hours | PH | E: Requirements of a school backpack: size, capacity and for its adequate transport  C: Control | The experimental group obtained statistically significant higher scores than the control group for the behaviour in the posttest and follow-up | Some concerns |
| Hinckson et al., 2013 | Quasi-experimental, New Zealand | 30 subjects  Age: 9-10  E = 23  C = 7 | 4 weeks | Standing workstation in classroom | E: Standing workstations in classroom  C: Control | The experimental group obtained statistically significant higher scores than the control group for the standing time, no differences were found in time spent walking and step count and control group obtained not statistically significant higher scores in the sitting time and sit-to-stand counts in the posttest | High |
| Mayorga-Vega et al., 2013 | RCT, Spain | 72 subjects  Age: 11.1 (10-12)  E = 35  C = 37 | 8 weeks, 1.66 hours per week, 11.66 total hours | Exercise + PA  (Progressive) | E: Racing games + strengthening + hamstrings and lumbar stretching  C: Traditional games, basketball and volleyball activities | The experimental group obtained not statistically significant higher scores than the control group for the trunk flexion endurance, upper limbs endurance and cardiovascular endurance in the posttest | Some concerns |
| González-Gálvez et al., 2014 | Quasi-experimental, Spain | 57 subjects  Age: 14.11  E = 30  C = 27 | 6 weeks, 1.83 hours per week, 11 total hours | Exercise (Progressive) | E: Stretching exercises + strengthening + breathing exercises  C: Usual physical education classes (no specific) | The experimental group obtained statistically significant higher scores for the trunk flexion endurance and trunk extension endurance than the control group and not statistically significant higher scores for the hamstring flexibility than the control group in the posttest | High |
| Sellschop et al., 2015 | RCT, South Africa | 127 subjects  Age: 13.4  E = 61  C = 66 | 1 week, 0.75 hours per week, 0.75 total hours | PH | E: Carrying a school bag correctly, co-operative group work with problem–solving tasks related to poor postural habits and computer work  C: Control | The experimental group obtained not statistically significant higher scores than the control group for the behaviour in the posttest and statistically significant higher scores in the follow-up | Some concerns |
| Ritter & de Souza, 2015 | Quasi-experimental, Brazil | 49 subjects  Age: 14.69  E = 26  C = 23 | 10 weeks, 1.66 hours per week, 16.66 total hours | PH + Exercise + PA | E: Evolution of man and his spine, the emergence of spinal curves in humans: from birth to adulthood, role of spinal curves and spine structures + Sitting, standing, rising from a chair, sitting from writing, picking up objects from the floor and carrying schoolbags + Stretching + Recreational and associative activity  C: Control | The experimental group obtained statistically significant higher scores than the control group for the behaviour and posture in the posttest and not statistically significant higher scores for the behaviour in the follow-up, not statistically significant higher scores in the perception in the posttest and not statistically significant lower scores in the perception in follow-up | High |
| Brzek & Plinta, 2016 | Quasi-experimental, Poland | 366 subjects  Age:  E = 144  C = 222 | No specific | PH | E: Anatomy and function of the spine, causes of postural disorders, how bad postures may affect as adults, ergonomics in daily activities, weight of school bag + Good positions to carry the school bag (theoretical and practical)  C: Control | The experimental group obtained statistically significant higher scores than the control group for the behaviour and posture in the posttest | High |
| Dullien et al., 2018 | RCT, Germany | 176 subjects  Age: 10.55 (10-12)  E = 90  C = 86 | 12 weeks, 0.31 hours per week, 3.72 total hours | PH + Exercise | E: Anatomy + good and bad posture while sitting, healthy backpacks habits, healthy lifting and carrying, back-friendly sports and nutrition + stretching + strengthening of back (hip lifts and ball exercises) and abdominal muscles (plank, crunch and ball exercises). (Additionally, the use of posters on posture awareness, strengthening and stretching exercises)  C: Control | The experimental group obtained statistically significant higher scores than the control group for the behaviour, knowledge and posture, not statistically significant in the balance and the control group obtained not statistically significant higher scores in the upper limb endurance and statistically significant higher scores in the trunk flexion endurance in the posttest | Some concerns |
| Sellschop et al., 2018 | RCT, South Africa | 127 subjects  Age: 13.4  E = 61  C = 66 | 1 week, 0.75 hours per week, 0.75 total hours | PH + Exercise | E: Posture, backpack weight + workstation set-up + neck, shoulders and lower back stretches  C: Control | The experimental group obtained statistically significant higher scores than the control group for the behaviour in the posttest | Some concerns |
| González-Gálvez et al., 2019 (a) | RCT, Spain | 52 subjects  Age: 14.44 (14-16)  E = 26  C = 26 | 6 weeks, 1.83 hours per week, 11 total hours | Exercise (Progressive) | E: Stretching of back and hamstrings muscles + strengthening + pelvis retroversion and anteversion + breathing + relaxation + raquis flexion, extension, rotation and hip mobilization + self-massage of the spine  C: Endurance and strengthening exercises based on running, strength circuits, plyometrics, or sport/cooperation games | The experimental group obtained statistically significant higher scores for the trunk flexion endurance and not statistically significant scores in the trunk extension endurance and hamstring flexibility than the control group in the posttest | Some concerns |
| González-Gálvez et al., 2019 (b) | RCT, Spain | 101 subjects  Age: 14.52  E = 81  C =20 | 6 weeks, 1.83 hours per week, 11 total hours | Exercise (Progressive) | E: Stretching exercises + abdominal, lumbar exercises and glute exercises + breathing techniques + correct segmental placement  C: Usual physical education classes | The experimental group obtained statistically significant higher scores than the control group for the trunk flexion endurance and trunk extension endurance in the posttest | Some concerns |
| Kiss et al., 2019 | RCT, Hungary | 137 subjects  Age: 15.38  E = 68  C = 69 | 24 weeks, 2.5 hours per week, 60 total hours | PA + Exercise (Progressive) | E: Stretching + back muscles strengthening, lumbar motor control + correct posture in standing and sitting position + static exercises on instable surface + specific exercises (kayak or canoe)  C: Control | The experimental group obtained statistically significant higher scores than the control group for the trunk flexion endurance and lumbar motor control in the posttest | Some concerns |
| Miñana-Signes et al., 2019 | Quasi-experimental, Spain | 32 subjects  Age: 11.16 (10-12)  E = 16  C = 16 | 2 weeks, 2.62 hours per week, 5.25 total hours | PH + Exercise + PA | E: Spine teaching + correct and incorrect postural habits + stretching + strengthening the trunk musculature + pelvis lift + postural correction + relaxation (Jacobson) + football and floorball + racing games  C: Usual physical education classes | The experimental group obtained statistically significant higher scores than the control group for the knowledge in the posttest and behaviour in the posttest and follow-up | High |
| Batistão et al., 2019 | Quasi-experimental, Brazil | 282 subjects  Age: 11.5  E = 188  C = 282 | 8 weeks, 1.66 hours per week, 13.33 total hours | Exercise + PA | E: Stretching + concentric and isometric exercises + aerobic sports + aerobic games  C: Control | The experimental group obtained not statistically significant higher scores than the control group for the posture in the posttest | High |
| Akbari-Chehrehbargh et al., 2020 | RCT, Iran | 104 subjects  Age: 11  E = 52  C = 52 | 6 weeks, 1 hour per week, 6 total hours | PH + Exercise | E: Spine teaching + backpack wearing, carrying objects, proper sitting and standing postures + back strengthening + stretching | The experimental group obtained statistically significant higher scores than the control group for the behaviour, knowledge, self-efficacy, skills and beliefs in the posttest and follow-up | Some concerns |

^RCT: randomized controlled trial, PH: postural hygiene, PA: physical activity^
