# Supplementary material for: Effectiveness of physiotherapy interventions for back care and the prevention of non-specific low back pain in children and adolescents: a systematic review and meta-analysis
Source: BMC Musculoskelet Disord. 2022 Apr 2;23:314. doi: 10.1186/s12891-022-05270-4 (PMC8976404; doi:10.1186/s12891-022-05270-4)
Supplement: Supplementary file 5 — Additional file 5. [file 12891_2022_5270_MOESM5_ESM.docx]

**Author(s)**: José Manuel García Moreno, Inmaculada Calvo Muñoz, Antonia Gómez Conesa, José Antonio López López

**Question**: Preventive physiotherapyany treatment compared to control for back care in children and adolescents

**Setting**:

**Bibliography**:

| **Certainty assessment** | | | | | | | **№ of patients** | | **Effect** | | **Certainty** | **Importance** |
| --- | --- | --- | --- | --- | --- | --- | --- | --- | --- | --- | --- | --- |
| **№ of studies** | **Study design** | **Risk of bias** | **Inconsistency** | **Indirectness** | **Imprecision** | **Other considerations** | **Preventive physiotherapyany treatment** | **control** | **Relative (95% CI)** | **Absolute (95% CI)** |  |  |
| **Back care related behaviour (assessed with: schoolbag weight, behaviour in daily life test, behaviour in a trial test)** | | | | | | | | | | | | |
| 9 | randomised trials | serious ^a^ | not serious | serious ^b^ | not serious | publication bias strongly suspected very strong association ^c^ | 924 | 819 | - | SMD **1.19 SD higher** (0.62 higher to 1.76 higher) | ⨁⨁⨁◯ MODERATE | CRITICAL |
| **Back care related knowledge (assessed with: knowledge questionnaires)** | | | | | | | | | | | | |
| 4 | randomised trials | serious ^a^ | not serious | serious ^b^ | not serious | publication bias strongly suspected very strong association ^d^ | 361 | 355 | - | SMD **1.84 SD higher** (1.04 higher to 2.63 higher) | ⨁⨁⨁◯ MODERATE | IMPORTANT |
| **Trunk flexion endurance (assessed with: Kraus-Webber test, sit-ups 30 secs, bench trunk curl test)** | | | | | | | | | | | | |
| 7 | randomised trials | serious ^e^ | not serious | serious ^b^ | not serious | publication bias strongly suspected strong association ^c^ | 358 | 295 | - | SMD **0.65 SD higher** (0.1 higher to 1.2 higher) | ⨁⨁◯◯ LOW | IMPORTANT |
| **Trunk extension endurance (assessed with: Sorensen test)** | | | | | | | | | | | | |
| 4 | randomised trials | serious ^f^ | not serious | not serious | not serious | publication bias strongly suspected strong association ^d^ | 165 | 103 | - | SMD **0.71 SD higher** (0.44 higher to 0.98 higher) | ⨁⨁⨁◯ MODERATE | IMPORTANT |
| **Posture (assessed with: new york posture rating, observation of the seated posture in the classroom, measurement of the angle of kyphosis and lordosis, Matthiass test, postural evaluation software)** | | | | | | | | | | | | |
| 5 | randomised trials | very serious ^g^ | not serious | serious ^b^ | not serious | publication bias strongly suspected strong association ^d^ | 517 | 484 | - | SMD **0.65 SD higher** (0.34 higher to 0.97 higher) | ⨁◯◯◯ VERY LOW | IMPORTANT |
| **Hamstring flexibility (assessed with: sit and reach, toe-touch test)** | | | | | | | | | | | | |
| 4 | randomised trials | serious ^h^ | not serious | not serious | not serious | publication bias strongly suspected ^d^ | 115 | 113 | - | SMD **0.46 SD higher** (0.19 higher to 0.72 higher) | ⨁⨁◯◯ LOW | IMPORTANT |

**CI:** Confidence interval; **SMD:** Standardised mean difference

#### Explanations

a. Some studies are non-randomised

b. Different tools are used to measure this variable, so there is no homogeneity in this section.

c. Meta-regression analysis with n as a covariate suggests publication bias.

d. Few studies included, no analysis of the risk of publication bias could be done.

e. A study is non-randomised and there is no blinding of evaluators in general.

f. A study has a high risk of bias

g. All but one of the studies have a high risk of bias.

h. Half of the studies have a high risk of bias
